# The Emotional Machiavellian: Interactions Between Leaders and Employees

**DOI:** 10.1007/s10551-022-05233-8

**Published:** 2022-09-07

**Authors:** Nilupulee Liyanagamage, Mario Fernando, Belinda Gibbons

**Affiliations:** Faculty of Business and Law, University of Wollongong, Wollongong, NSW Australia

**Keywords:** Machiavellian leadership, Emotions, Relational process, Global South

## Abstract

This paper examines the emotional processes in Machiavellian leadership. The leadership literature portrays Machiavellians as ‘dark’ individuals that engage in unethical actions, causing employee dissatisfaction, distress, emotional exhaustion and high turnover. However, research has seldom questioned the processes behind these unethical and negative outcomes. This study explores Machiavellian emotional processes at multiple levels—within-persons and relational levels (between-persons and interpersonal interactions in organisations). In this study, emotions and leadership are not explored in isolation but as social processes that occur in relationships between leaders and employees in evolving organisational settings. This study draws on 20 participants from four large multi-national construction firms in Sri Lanka. Open-ended semi-structured interviews were conducted to explore the emotions of Machiavellians in organisations. The findings suggest that Machiavellianism influences leader and employee emotional processes. Furthermore, the emotional processes, influenced by Machiavellianism, appear to facilitate the development of leader and employee relationships and emotional experiences at within-persons and relational levels in organisations.

## Introduction

In today’s increasingly uncertain business environment, leaders and employees must excel in organisational achievements and consider their actions from an ethical standpoint to successfully navigate the complex personal, organisational and public expectations. Many leaders and employees suffer from the unabated instances of ethical misconduct, manipulation and self-serving behaviour in organisations (Koo & Lee, 2021). Consequently, and not surprisingly, there is growing interest in the ‘dark’ side of organisational behaviour (Liang et al., 2021; Mackey et al., 2021), the Dark Triads and their influence on leadership (Lyons et al., 2019). Dark Triads comprise narcissism, psychopathy and Machiavellianism. The first two constructs have been explored in detail (Harrison et al., 2018; Mutschmann et al., 2021). Yet, Machiavellianism remains overlooked, especially in leadership studies. Machiavellians are commonly described as cynical and controlling individuals using amoral manipulation to achieve personal goals and status (Dahling et al., 2009).

Machiavellianism in organisations relates to unethical actions, employee dissatisfaction, distress, emotional exhaustion, turnover and other negative workplace consequences (Bagozzi et al., 2013; Belschak et al., 2018b; Den Hartog & Belschak, 2012). Although much research has considered the outcomes of Machiavellian leaders on employee performance, satisfaction, well-being and organisational goals (Belschak et al., 2018a; Castille et al., 2018; Koo & Lee, 2021), to our knowledge, no published study explicitly explores *why* Machiavellians engage in unethical actions in organisations.

To consider the *why*, in this research, we explore the relational processes of Machiavellian leadership –specifically the emotional processes. The interest in emotions in organisations (Ashkanasy & Dorris, 2017; Barsade et al., 2018), leadership (Humphrey et al., 2016; Silard & Dasborough, 2021), and personality traits (Nagler et al., 2014; Puthillam et al., 2021) is not novel. Research propose that individuals with high emotional stability are likely to adapt and cope with challenges, obstacles and demands in their workplace (Liu & Yu, 2019). Yet, the principle is that it is “inappropriate to express emotions” in organisations, especially among destructive leaders (and employees) who rely on their control of others; as such, they attempt to minimise uncontrollable emotions (Chiang et al., 2021, p. 1084). Since leaders and employees frequently experience obstacles in the workplace, it is important to explore the emotional processes of Machiavellians in relation to others.

This study can be distinguished from recent studies in Machiavellianism and emotions in leadership in several important ways. First, we explore the multiple levels of emotions at within-persons and relational levels (including between-persons and interpersonal interactions) in organisations, among leaders and employees with varying levels of Machiavellianism. We highlight various emotional processes of Machiavellian leaders, employees and dyads in different situations in natural workplace settings. Second, we contribute to follower-centric research (Goswami et al., 2020) by exploring employee Machiavellianism on leaders’ experiences. Although research has considered the effect of leader Machiavellianism on followers (Erkutlu & Chafra, 2019; Stradovnik & Stare, 2018), there is little discussion on the importance of follower Machiavellianism. We argue that attention to followers’ Machiavellianism is relevant to understanding leaders’ emotional experiences.

Third, literature on emotions primarily represents ‘Western’ cultures (Emmerling & Boyatzis, 2012; Miao et al., 2020). But emotional experiences and expressions of leaders and employees may differ in the Global South compared to the Global North due to cross-cultural and contextual differences. We examine Machiavellians’ emotional processes from a Global South perspective. Thus, we contribute to the literature on Machiavellianism, leadership, and emotions in the Global South. Last, we present the development of a framework for the emotional process of Machiavellians in organisations, with implications for theory and practice.

## Literature Review

Leadership is inherently an emotional process (Ashkanasy, 2003; George, 2000; Humphrey et al., 2016). Several scholars have recognised the importance of emotions to charismatic leadership (Sy et al., 2018), transformational leadership (Ashkanasy & Tse, 2000), authentic leadership (Agote et al., 2016) and leader–member relationships (Dasborough & Ashkanasy, 2002; Herman et al., 2018). Emotions are a mental response triggered by an event or entity (Izard, 1991). Ashkanasy (2003) proposes five levels of emotions in organisations: within-person, between-persons, interpersonal interactions, groups and organisation-wide. *Within-person* research explores how an individual’s emotions evolve in response to various events (Ashkanasy & Dorris, 2017). At this level, leadership scholars examine emotional labour in particular, which is the “management of feeling to create a publicly observable facial and bodily display…to induce or suppress feeling in order to sustain the outward countenance that produces the proper state of mind in others” (Hochschild, 1983, p. 7). Scholars further study how emotions affect leaders’ decision-making (Ashkanasy & Daus, 2002) and leaders’ capacity to empathise (George, 2000), that is, the capacity to understand another’s feelings and re-experience them.

*Between-persons* research explores emotions through individual differences in leader emotions (Marroquín et al., 2016) and the ability to manage one’s feelings—emotional regulation (George, 2000). Through emotional regulation, individuals attempt to influence their emotional experiences, when and how they are experienced and expressed (Gooty et al., 2010). Emotional regulation consists of the experience of emotion (felt emotion) and expression of emotion (displayed emotion). In organisations, how a leader feels and regulates emotions, whether through intensification, maintenance or suppression, ultimately determines how they show emotions (Braunstein et al., 2017). The ability to express emotions can influence how subordinates perceive a leader and form effective leader–member relationships (Dasborough & Ashkanasy, 2002; Graen & Uhl-Bien, 1995). However, the energy and capacity of emotional regulation can cause emotional exhaustion, that is, “depleted emotional energy and resources and the resulting physical and mental fatigue” (Stradovnik & Stare, 2018, p. 1038).

At an *interpersonal interactions* level, leadership studies concentrate on the emotions of group members: the spread of emotions through a group (i.e. emotional contagion) (Ashkanasy & Dorris, 2017) and emotions in dyadic partnerships (Herman et al., 2018). At this level, emotions are studied not in isolation but with the understanding that multiple emotions can be experienced simultaneously. Leaders may try to induce and spread emotions through a group (Humphrey et al., 2016; Tee, 2015). Emotion contagion affects group performance, outcomes and members’ experience of emotions (Sy et al., 2005). Leaders who transfer positive moods positively affect group members, whereas negative emotional transfer can create negative emotions in members (Barsade et al., 2018). *Groups* and *organisation-wide* levels view leadership as a social process that influences the moods and feelings of group members (Ashkanasy & Dorris, 2017). In this stage, mood management is a critical element of team leadership (Ghosh et al., 2012).

Despite the extant literature on emotions and leadership and the importance of emotions to leadership emergence (Côté et al., 2010), leader effectiveness (Tee, 2015), leader–follower relationships (Dasborough & Ashkanasy, 2002), performance (Chi & Ho, 2014) and outcomes (Lindebaum et al., 2017), there is still considerable ambiguity and limited research on the understanding of emotions in leadership and organisations (Humphrey et al., 2016; Silard & Dasborough, 2021). This is particularly evident in the lack of research on the role of emotions in interpersonal interactions, groups and organisation-wide. Both emotions (Ashkanasy & Dorris, 2017) and leadership do not occur in isolation (Cunliffe & Eriksen, 2011; Graen & Uhl-Bien, 1995), and research must reflect the relational nature of these social processes.

### Dark Leadership and Emotions

Primarily research has focused on the benefits of emotions in leadership (Côté et al., 2010; Lone & Lone, 2018). However, the outcomes/consequences of emotions in leadership can be prosocial and non-prosocial (Austin et al., 2007, 2014). Various emotional management or regulation strategies can result in better or worse outcomes (Nagler et al., 2014; O’Connor & Athota, 2013). Ideally, a leader should attempt to promote ethical conduct and guide employees through ethical dilemmas (Brown & Mitchell, 2010). Emotions can consciously and unconsciously influence ethical decision-making and behaviours (Salvador & Folger, 2009). However, leaders can also promote negative emotions among followers (Chi & Ho, 2014), which can spread throughout the organisation, hurting employee experiences and forming cynical perspectives towards the leader (Tee, 2015). Therefore, the role of emotions is not only relevant for effective styles of leadership but also important to furthering research on destructive and ‘dark’ leadership.

Scholars have studied (negative) emotions in the Dark Triad (Austin et al., 2014), emotions in authoritarian leadership (Chiang et al., 2021) and unethical leadership (Brown & Mitchell, 2010). There have only been a limited number of studies on Machiavellianism and emotions (Austin et al., 2007, 2014; O’Connor & Athota, 2013), and even fewer on Machiavellian leadership and emotions (Gkorezis et al., 2015; Stradovnik & Stare, 2018). To address the evident gap in research, this paper explores the emotional processes in Machiavellian leadership.

### Machiavellian Leadership and Emotions

Machiavellian leadership is “viewed as cynical and unprincipled in its use of intentionally manipulative behaviours to elicit cooperation and compliance from followers” (Muenjohn et al., 2018, p. 81). They “focus on power and control, and the development of personal relationships only with those who have the power or influence to help them succeed” (Guillet et al., 2012, p. 201). Machiavellian leaders assert control over subordinates by influencing their salaries, promotions, rewards and punishments (Guillet et al., 2012). Consequently, it creates an absence of trust between the leader and follower, leading to increased organisational cynicism among subordinates due to negative emotions and emotional exhaustion (Gkorezis et al., 2015; Stradovnik & Stare, 2018).

Nevertheless, few scholars find that Machiavellians can perceive emotions of others and engage in emotional sharing (Austin et al., 2007; Bagozzi et al., 2013). They observe that high Machiavellians can resonate empathically than low Machiavellians. Nichols (2001) suggests that Machiavellians’ ability to be successful rests, at least partially, in their skills to understand others’ perspectives and their ability to “explain and predict behaviour” of interacting partners (Langdon, 2003, p. 240). Machiavellian leaders can use the knowledge gained from their sense of empathy to understand and positively influence followers’ emotions and attitudes, to support corporate goals.

But in some situations, Machiavellians ability to empathise may aid them emotionally manipulate others (Austin et al., 2007; Bagozzi et al., 2013). Andrew et al. (2008) suggest that Machiavellianism is the opposite cognitive style of empathy. Other studies also show a negative correlation between Machiavellianism and empathy, or empathy-like traits (Bagozzi et al., 2013; Wastell & Booth, 2003), indicating they are unsupportive, selfish and insensitive leaders with little regard for others (Sakalaki et al., 2007). High Machiavellian employees in unethical leadership settings are likely to exhibit increased knowledge hiding and emotional manipulation (Belschak et al., 2018a).

The review of the literature identifies several areas for theory development. Although there is a large body of extant literature on emotions in leadership (Humphrey et al., 2016; Sy et al., 2018), they are primarily focused on effective leadership styles and lesser on the role of emotions in unethical leadership (Brown & Mitchell, 2010). However, there is a clear shift in scholarly interests towards negative leadership (Koo & Lee, 2021) and consequently towards Machiavellian leadership (Belschak et al., 2018a) and Machiavellian emotions (Gkorezis et al., 2015; Stradovnik & Stare, 2018). Yet, there are several gaps to address. First, the effectiveness of Machiavellian leadership has been evaluated based on leadership outcomes rather than the relational processes of the leadership (Uhl-Bien & Ospina, 2012). Second, research on Machiavellian emotions is primarily at a *within-person* level with some references to *between-persons* implications. There are significant theoretical implications in uncovering the relational aspects of Machiavellian emotions, such as the process of emotional contagion or emotion regulation in relationships. Third, Machiavellian leadership studies have often relied on a WEIRD (i.e. White, Educated, Industrialised, Rich, and Democratic) sample in non-organisational settings (Puthillam et al., 2021). Leadership scholars have called for studies of leaders and followers in natural organisational settings to explore the relational aspects of leadership (Cunliffe & Eriksen, 2011; Uhl-Bien & Ospina, 2012).

We explore the emotional processes in Machiavellian leadership. Accordingly, we will address the calls to examine emotions in negative leadership (Brown & Mitchell, 2010), leadership as a relational process (Uhl-Bien & Ospina, 2012), and the emotions in *within-person* as well as *between-persons* and *interpersonal interactions* at the organisational level (Ashkanasy, 2003; Barsade et al., 2018; Herman et al., 2018). The following section will discuss the study context and the research methodology.

## Machiavellianism, Leadership and Emotions in the Global South

This study takes place in the Global South. Accordingly, the review of literature is led by considering cross-cultural implications. Research shows that emotions associate differently in the Global South compared to the Global North (Hafen et al., 2011). Different cultures with different languages may use different words to describe emotions (Emmerling & Boyatzis, 2012). Some cultures may have an extended vocabulary to convey emotion than others. There may be cultural differences in the intensity of experience and expression of emotions (Caruso, 2008). However, most study populations represent ‘Western’ cultures, such as the UK, USA and Australia (Emmerling & Boyatzis, 2012). Therefore, it is essential to question whether constructs such as emotions and leadership have different meanings in the Global South (Lone & Lone, 2018).

Furthermore, there are cultural implications for the Machiavellian concept. The term ‘Machiavellian’ was based on *The Prince* by Niccolò Machiavelli, an Italian political advisor. It was later adopted by American psychologists Christie and Geis (1970). The concept and measures of Machiavellianism originate from the Global North, so it may not project the same meanings in the Global South. There have been some cross-cultural studies on Machiavellianism (i.e. Shafer & Wang, 2011; Siu & Tam, 1995), but only a few have examined the conceptual equivalence of the concept across cultures (Kuo & Marsella, 1977). While Machiavellianism can adequately measure the personality of Americans, it is unreasonable to assume that it is conceptually equivalent to other cultures.

This study acknowledges cultural differences and that conceptual differences may arise with applying Global North origin constructs to the Global South. The following sections introduce the research context, with precedence to cross-cultural rationale.

### Sri Lanka

Sri Lanka is an island in South-East Asia, with a population of 21.8 million. It is a multi-religious, multi-ethnic and multi-linguistic nation. Sri Lanka boasts a rich history, starting from the origins of the Sinhalese people, recorded in the *Mahavamsa* (the ‘Great Chronicle’). The story of King Dutugemunu (161_BCE_ to 137_BCE_) had a substantial impact on Sinhalese history when he overthrew Tamil King Elara to recapture Anuradhapura (ancient Sinhalese capital) (Kapferer, 2011). Mahavamsa (1912, p. 178) writes about millions of Tamil people slain in the war by King Dutugemunu, but “this deed arises no hindrance in thy way to heaven. Only one and half-human beings have been slain here by thee”. *Mahavamsa* articulates that King Dutugemunu will undoubtedly go to heaven because he only killed one and a half-human beings: a mother and her unborn child. The millions of Tamil deaths are not considered a sin but rather a sacrifice by the King to reclaim his kingdom.

Western literature considers Sri Lanka a collectivist nation with strong family values and a feminine culture with emotional, caring and sympathetic people (Hofstede, 2011). Nevertheless, it is vital to consider that in historical Sri Lanka, evil acts are accepted to reinstate the righteous Buddhist state. Even today, King Dutugemunu is not regarded as evil or wicked; but perceived as an “ideal king” and hero (Kapferer, 2011). What these ideologies mean in terms of Machiavellianism, leadership, and emotions is yet to be considered in academic literature.

There is an abundance of text about Sri Lankan people by Sri Lankan people in the Sinhalese language. Yet, there are only a few published articles from credible peer-reviewed journals on the leadership and emotions of the Sri Lankan people (Fernando & Jackson, 2006; Perry et al., 2015). We consider Sri Lanka not as a *unique* context but as a *significant* research context. This study applies theories from the Global North to the Global South. The implications of this study are not only relevant to Sri Lanka, but for countries with growing migrant Sri Lankans in prominent leadership positions, other similar developing countries deprived of a platform in the Global North literature, and, more importantly, cross-cultural literature.

## Study Context: Construction Industry in Sri Lanka

This study is set in the Sri Lankan construction industry. This industry is important to economic growth in developed and developing countries. However, the construction industry faces numerous ethical challenges (Ho, 2011). These ethical issues are amplified in developing contexts such as Sri Lanka, where construction firms struggle due to scarcity of essential resources, lack of skilled workers, political influence, uncertain economic conditions, and restrictive trade policies (World Bank, 2018). To survive, construction firms prioritise profitability and achievement of goals over ethical practices (Lu et al., 2016). Leadership is pivotal in how organisations and leaders act in competitive and ethically compromising situations.

The construction industry is transitionary and project-based. This means that firm structures are temporary, and teams will form, disband and reform to shift to a new project. Relationships within construction sites have limited time to develop before they accomplish the project and change to another project. The challenges of operating in transitionary project-based environments are distinct from stable environments (Loosemore et al., 2020). Construction firms in developing countries are challenged with both organisational and country-level uncertainties. Yet, leadership research in construction industries (Graham et al., 2020) in developing countries (Yap et al., 2019) is scarce.

Research suggests that construction project managers are focused on the “end goals and not the means” (Ofori, 2008, p. 620). Consequently, their employees perceive project managers as ‘managers’ but not as ‘leaders’ (Russell & Stouffer, 2003). Furthermore, relationships within construction sites are influenced by highly masculine ideologies (Liyanagamage & Fernando, 2022). It is one of the most male-dominated trades (George & Loosemore, 2019). The actions and behaviours of those in masculine cultures are associated with stereotypical male behaviours. These include the suppression and denial of emotions, aggression and competitiveness, and physical strength (Naoum et al., 2020).

This study seeks to understand an important social process: emotional processes in leadership, specifically ‘dark’ Machiavellian leadership in the Sri Lankan construction industry. The rich context of this study provides important direction for literature on emotions, leadership and Machiavellianism. First, developing countries are rarely considered in studies of Machiavellianism, yet uncertain national and organisational contexts in developing contexts have important implications for leadership (Yap et al., 2019). Second, transitionary project-based organisational structures are seldom considered in leadership research (Giritli & Oraz, 2004). Third, the highly masculine nature of the construction industry has important implications on emotions and leadership (George & Loosemore, 2019). In this research, we consider the construction industry in a developing country, an important yet neglected context, to highlight the emotional processes in Machiavellian leadership.

## Methodology

This study uses qualitative research methods to explore the emotional processes in Machiavellian leadership in four construction organisation sites in Sri Lanka. Qualitative research is beneficial for exploring complex organisational processes and especially appropriate for studies investigating leadership processes (Judge et al., 2002; Uhl-Bien & Ospina, 2012) and emotional processes (Ashkanasy & Dorris, 2017; Puthillam et al., 2021).

### Sample

A purposive sampling strategy (Patton, 2002) was used to identify the firms and the research sample. Twenty participants were drawn from different hierarchical levels of four multi-national construction firms operating in Sri Lanka. We focused on a smaller yet practical sample size (Mason, 2017) to conduct a deep analysis (Sandelowski, 1995) that is attentive to nuances in individuals, context and emotions in leadership (Guest et al., 2006). Table 1 presents participant demographic and organisation information. Identifiable information has been removed for confidentiality. All sample organisations are large-scale construction firms operating on multiple sites. The four sites presented in this study are based in Colombo, with over 200 employees in each site and projects worth around 1–4 million USD. Sites 1 and 2 have a high Machiavellian (high Mach) leader, and Sites 3 and 4 have a low Machiavellian (low Mach) leader. The overall sample consists of five high Machs and 15 low Machs. The average age of the sample is 37 years. This sample consists of 18 male and two female participants. The highest education qualification is Chartered Engineer, followed by bachelor’s degree, advanced/higher diploma, diploma and Advanced level (Year 13) qualifications.

**Table 1:** Demographic and organisational information of participants (leaders and employees) in construction sites (*N* = 20)

Site no	Leader/ employee status	Name	Age	Gender	Highest level of education	Machiavellian Personality*	Organisational position	Relationship with leader
1	Leader	Ian	41	Male	BSc Civil Engineering	High Mach	Project manager	-
	Employee	Charles	32	Male	National Vocational Qualification	Low Mach	Construction manager	3 years
	Employee	Travis	30	Male	BSc Civil Engineering	High Mach	Project engineer	5 years
	Employee	Anton	51	Male	Diploma	High Mach	Mechanical foreman	3 years
	Employee	Patrick	47	Male	Advanced Level	Low Mach	Technical officer	1 year
	Employee	Vivian	28	Female	Diploma in Building Construction	Low Mach	Supervisor	1 year
2	Leader	Uday	52	Male	BSc Civil Engineering	High Mach	Project manager	–
	Employee	Claire	34	Female	BSc Civil Engineering	Low Mach	Quality assurance and control Engineer	2 years
	Employee	Chris	37	Male	Diploma in Building	Low Mach	Civil engineer	1.5 years
	Employee	Pravish	27	Male	National Diploma in Technology	Low Mach	Assistant mechanical engineer	2 years
	Employee	Mike	29	Male	Higher Diploma in Quantity Surveying	Low Mach	Assistant quantity surveyor	1.5 years
3	Leader	Stuart	53	Male	Charted Engineer	Low Mach	Project director	–
	Employee	Ivon	42	Male	BSc Mechanical Engineering	Low Mach	Engineer	2 years
	Employee	Bernard	34	Male	Charted Engineer	Low Mach	Engineering manager	4 years
	Employee	Kenny	43	Male	National Diploma	Low Mach	Site manager	2 years
	Employee	Paul	26	Male	BSc Civil Engineering	Low Mach	Planning engineer	2 years
4	Leader	Thomas	51	Male	Chartered Engineer	Low Mach	Ass gm construction	–
	Employee	Brian	31	Male	BSc Civil Engineering	High Mach	Civil engineer	6 years
	Employee	Kalva	33	Male	BSc Civil Engineering	Low Mach	Quality assurance and control Engineer	1 year
	Employee	Aziz	26	Male	BSc Civil Engineering	Low Mach	Civil engineer	1.5 years

^*^Machiavellian personality of participants was measured using the MACH IV survey by Christie and Geis (1970). High Machs scored 60 and over; low Machs scored less than 60 in the MACH IV survey

### Data Collection

This research employed a semi-structured interview technique to offer insightful examples (Alvesson & Sveningsson, 2003), and illuminate contextual elements (Bryman et al., 1996). Although quantitative surveys are more dominant in personality and leadership studies, they are static and impose ‘preconceived conceptual schemas’ (Bryman et al., 1988). Likewise, there is ambiguity around applying quantitative assessments of emotions (such as Emotional Intelligence scales) to populations outside the Global West (Emmerling & Boyatzis, 2012). Qualitative studies are helpful to examine the dynamic and processual nature of emotions (Gooty et al., 2010) while revealing contextual aspects of emotional processes. Interview accounts will add richness to the context, explanations and diversity of perspectives.

An interview protocol was developed to guide the interviews based on a priori literature on emotions and leadership. To examine the *within-person* and *between-persons* levels of emotions in leaders and followers, we questioned the participants on (i) their perspectives on expressing emotions in the workplace, (ii) coping with negative or positive emotions, (iii) the role of emotions in decision-making and (iv) their knowledge about the impact of emotional expressions in the workplace (i.e. consequences of emotions). To examine the relational dimensions, that is, *between-persons* emotions and *interpersonal interactions* at the organisational level, we question the participants on (i) how their leader/employee makes them feel (i.e. emotion contagion), (ii) their expressions of emotions in relationships and (iii) their sharing of emotions with others in the organisation (i.e. emotional sharing). Some open-ended questions allowed participants to reflect and express their feelings and perceptions freely. The interviews lasted between 20 and 60 min and were conducted face-to-face.

Interview data were digitally recorded and transcribed. Interviews conducted in Sinhalese were translated during the transcription process by the first author with the support of the second author, both of whom are bilingual (English and Sinhalese). The translation process included a combination of literal (i.e. word for word translation) (Wagner-Tsukamoto, 2009) and free translation (i.e. sense for sense translation) to try to be as true to the meanings conveyed by the participants. The authors checked the translated transcripts against the recorded interview to improve the conceptual equivalence (Cha et al., 2007) and ensure ease of understanding for an English-speaking audience.

### Data Analysis

The interview data analysis drew on the thematic analysis technique by Braun and Clarke (2012), which helped systematically identify, organise, and develop insights into patterns of meaning in the data. The analysis process employed a hybrid approach (Braun & Clarke, 2012). It included deductive coding as we drew on theoretical constructs of emotional levels in organisations (Ashkanasy, 2003), relational leadership (Uhl-Bien & Ospina, 2012), and inductive thematic coding from the dataset. This analysis allowed us to identify relevant patterns to answer the research questions (Braun & Clarke, 2012). Initially, we carefully read and re-read the interviews to familiarise ourselves with the dataset while also referring to the field notes written by the first author during data collection. The next phase involved synthesising interview transcripts, generating codes from the data and searching for themes. Several patterns emerged around the key themes related to emotions and leadership processes. A codebook (Table 2) was developed to guide the data analysis process.

**Table 2:** A sample illustration of the coding framework developed from the thematic data analysis

Main theme	Sub-theme	Illustrate quote
Within-person level emotions		
Expressing emotions	Avoid expressing emotions	*In this field you cannot be emotional. Cannot be… (*Thomas*)*
	Express negative emotions	*I feel very angry…it hurts…(*Travis*)*
	Express positive emotions	*I’m trying to be happy and keep my team happy… (*Ian*)*
Coping with emotions	Suppression	*I will try to manage, I will bear their scolding (*Travis*)*
	Mood management	*[It is] stressful…I try not to take on the stress (*Ivon*)*
	Display anger	*I’ve informed my problems… I’m very surprised how [others] can bear it all and remain silent. I just can’t do that…. (*Anton*)*
Consequences of emotions	Affect the mind	*We will have to suffer for it… (*Vivian*)*
	Well-being	*I can’t always be a guy who is tough, because then I will suffer and that will affect my health. (*Stuart*)*
	Consequences on others	*If I get angry, that’s not good for me, [and] not [good] for them… (*Ian*)*
Relational level emotions (i.e. between-persons, interpersonal interactions at organisational level)		
Emotional contagion	Positive spread of emotions	*At the end of the day all parties should be happy… (*Uday*)*
	Negative spread of emotions	*My leadership is that I want to act as a friend… (*Travis*)*
	Emotional sharing	*I have friends, they are also project managers, I’m asking them, and they are also in the same situation. (*Travis*)*
Impact of emotional contagion	Employee positive emotions	*I honestly think there is good leadership …there is good teamwork… (*Patrick*)*
	Employee negative emotions	*Honestly, we’re scared… (*Vivian*)*
	Distrust	*Out in the open, they are nice and friendly, you feel happy to work but there are things you see beneath the surface, then in the deep you see what really happens. (*Anton*)*
	Trust	*Trust him to some extent (*Travis*)*
Contextual level		
Organisational	Organisational hierarchy	*“Higher [up the hierarchy] I go, I keep thinking those people are right… (*Mitch*)*
	Office setting	*There are glass rooms, and bosses are in those rooms… they will never sit with us and talk normally…(*Vivian*)*
National	Respect	*We respect leaders, and we listen to them because respect is always in our nature… (*Claire*)*
	Power distance	*I think of the gap, the level gap…they think he’s so above them… (*Travis*)*
	Religion	*Buddha said… If you always get stressed and angry, then that will show in your face, then your body, soul and everything will suffer for nothing. (*Stuart*)*

## Emotional Processes of Machiavellians

Emotions in leadership are experienced at personal and dyadic levels in evolving organisational situations – and this is no exception to Machiavellian leadership. The following discussion examines the emotional experiences of Machiavellians at the within-person level and relational level (that is, between-persons and interpersonal interactions at organisational levels).

### Emotional Experiences of Machiavellians: Within-Person Level

To explore ‘*within-persons’* emotions in Machiavellian leadership, we asked leaders and employees to reflect on their emotional experiences in the workplace. The findings for within-persons level emotions in Machiavellian leadership are presented in three themes: emotions are for the weak, coping with negative emotions, and leveraging emotions to achieve goals.

#### Emotions are for the Weak

In the interviews, leaders spoke about the negative aspects of emotions, primarily referring to expressing emotions. The interviews show that Machiavellian leaders believe that expressing emotions in the workplace is a sign of weakness. As leaders, they are expected by themselves and others to be “strong”. As described by Ian, a high Mach leader:

The [General Manager] said to me “don’t follow your heart”. He said, “Don’t let your heart get ahead” …I must be strong. In the company, there is a company rule, not [to be] emotional… As a manager, sometimes I must be strong. [With] emotions, I can’t do work.

During the interview, Ian was asked how he thought the staff perceived him, for which initially he responded by asking, “Me?” with a bewildered expression. When asked again, Ian responded, “I’m not thinking [about] that! Any person can think anything. I’m not considering it…That does not matter to me”. This exchange indicates that perhaps high Mach leaders may avoid emotional experiences in the workplace to be perceived as “strong”. Low Mach leaders showed a similar understanding of emotions. Thomas explains that emotions are detrimental to a successful career in the construction industry. According to Thomas, there is a limit to emotions.

In this field you cannot be emotional. We cannot succeed being emotional…Emotional means taking care of people, we are emotional but within the requirement… our hearts are wet, but not externally…

The interviews with high Mach and low Mach leaders indicate a desire to avoid displaying emotions in the workplace because of their expectations and pressures from others (i.e. higher authorities and employees).

#### Coping with Negative Emotions

Workplaces can be stressful and exhausting. According to studies, individuals in transient and dynamic industries such as the construction industry may have to cope with many stressors. The interviews show that Machiavellian leaders and employees experience complex negative emotions in the workplace. However, their strategies to cope with those negative emotions may depend, among other factors, on their Machiavellianism. High Mach employee, Travis, explains that sometimes Ian or Charles would visit the site and blame him for issues in the construction process. He notes feeling hurt but dealing with his emotions without expressing them.

I feel very angry. When the top management visits the site, if they spot a problem or labour idling, they will scold me on the spot…it hurts... but I will try to manage; I will bear their scolding…I have friends; they are also project managers, and they are also in the same situation.

Travis explains sharing his emotions to friends outside his workplace, yet working in the construction industry. Travis may refrain from expressing his feelings to colleagues in his workplace to avoid showing weakness. Perhaps he is coping with his emotions (internally) because a display of emotions would affect how others perceive him.

From a leader’s perspective, Ian notes that employees do not work hard and are selfish. Yet, he explains that he dislikes expressing negative emotions because it will affect his mood and employees. Rather than expressing negative emotions, Ian copes with negative emotions by “joking” with the employees about doing more work.

I don’t like to shout at people…I want to work with them friendlily. Especially for me, if I get angry, that’s not good for me, [and] not [good] for them… I’m trying to be happy and keep my team happy… [But] they’re not working hard… [They are] selfish… If I scold or scream at them [it won’t affect them because] that is their behaviour… I just joke with them and ask them to concentrate on the work…I feel awful, but I don’t tell them.

Although Ian explains that his emotional experiences are ‘bad’ for him, he does not want to express or share them with employees. Like Travis, Ian notes sharing his experiences with colleagues outside the workplace.

Stuart, a low Mach leader’s approach to coping with negative emotional experiences and his understanding of emotions differ from high Machs. He explains that negative emotional experiences that lead to stress are like a “poison”. From a Buddhist perspective, this poison can affect a person’s physical and mental health. Stuart believes it is more effective to display ‘negative’ emotions (i.e. hiss like a snake) occasionally because it is not possible to be tough all the time. Although it is ‘difficult’ to regulate emotions, he believes he will ‘suffer’ without it.

Buddha said that if you blame, stress or argue with someone then you’re getting poison from others into your body. If you’re always stressed and angry, that will show in your face, your body, soul, and everything will suffer for nothing… [I’m like a] snake…who only hisses once in a while, but not always…I can’t always be tough, I will suffer, which will affect my health. I want to have a relaxed mind. It’s difficult, but you have to try.

Other low Machs also explain that the construction industry is stressful. But they believe it is vital to have boundaries in the workplace. Ivon explains that he values limitations and does not cross those boundaries regardless of organisational expectations. He notes that he tries to “stay relaxed” and does not face many problems.

[It is] stressful…I try not to take on the stress. That is one of my values; whether I’m working here or elsewhere, there is a limit to work, and I don’t go beyond that limit. I stay relaxed. Because of that, it’s not a problem for me…

Interviews show that high and low Machs experience negative emotions in the workplace. High Machs try to avoid negative emotions and manage them without displaying them to others because it may affect their projection of “strength” in the workplace. Low Machs understand that negative emotions are inevitable, and they try to manage them because they can be harmful like “poison”. Unlike high Machs, low Machs are not afraid to show emotions because it may affect their image, but because negative emotions have consequences on themselves and others.

#### Leveraging Emotions to Achieve Goals

The interviews show that Machiavellians understand the consequences of emotions and use this understanding to achieve goals that would help their career. High Mach leader Ian explains that he had financially funded suppliers when they needed money for personal reasons. Perhaps Ian can empathise with the suppliers, so he takes the risk. But he also notes that if the suppliers are unhappy, they might decide to find another company. Ian is responsible as the project manager for retaining suppliers, therefore, he leverages his understanding of the suppliers’ emotions to ensure they remain in his company.

Sometimes if [suppliers] have a funeral or something, we can’t get company cash…I give [my money] and settle [later], I can cover for them…. Otherwise, they might leave. I should keep them here… proper subcontractors can’t be found everywhere. If I’m getting that responsibility, then I can take that risk…

Vivian, a low Mach employee, explains that she refrains from expressing negative emotions towards labourers because they have more bargaining power. If she shouts at labourers, they may leave. Vivian explains this would cause her to “suffer”. Like Ian, she leverages her understanding of others’ emotions to manage her own emotions, ensuring that her career is not affected.

We can’t [shout] at labourers. Because labourers will work on this site today, tomorrow [they] may be on another site. Even if we accidentally shout, the workers [might] quit…then we will have to suffer for it.

Other Machiavellians s also share a similar understanding. For instance, Uday, a high Mach leader, explains that it is vital to “keep all parties in good relations…so that it is easy to run the project”. Travis, a high Mach employee, notes that he pretends to build relationships and share positive emotions with others, which projects trust, respect, and protection. However, in the interview, he said it is an ‘act’ to motivate employees to work harder.

My leadership is that I want to act as a friend; while I’m showing that I’m protecting them, they have to show me work…

Not all Machiavellians wants to achieve personal goals that further their career. Some explain that they use their emotional understandings to help others. A low Mach leader, Stuart describes a situation where a trusted worker was injured. He supported the worker from an official capacity and initiated a separate fund because they value their commitment to the company.

There was an accident where someone was injured badly, at that time we officially did whatever we had to do, other than that, because he’s not well enough to work we paid him the salary for that period. We try to help… I initiated that because [I] know that person’s background and value, [and] the period they worked with us…

The interview accounts of Machiavellians show that they use their understanding of emotions to achieve goals. These goals can be for personal and/or self-less means. Although Machiavellians understand emotions and consequences, they might leverage their understanding to gain personal achievements. Machiavellians’ decisions in the workplace may depend on their relationships with others in the situation. The following section will explore emotional experiences in relationships.

### Emotional Experiences of Machiavellians in Leader–Employee Dyads: Relational Level

To explore *relational* emotions in Machiavellian leadership, we present the leader and employee reflections on between-persons experiences and interpersonal interactions. We asked the participants to reflect on their emotional experiences in relationships/partnerships in the workplace. The findings for relational level emotions in Machiavellian leadership are presented in two dyads: high Mach leader–high/low Mach employee and low Mach leader–high/low Mach employee.

#### High Mach Leader and (High and Low) Mach Employee

This section explores the dyadic relationships between high Mach leaders and employees. Information on dyads is found in Table 1. The interviews suggest that high Mach leaders try to control the employees by evoking specific emotional experiences. Ian explains that he tells the employees that the project is failing by evoking negative emotions. He notes that when the workers feel that the project is profitable, they will try to “relax”, but when they think that the project will fail, they will be more focused – perhaps driven by fear of failure.

[My] staff know [that] this project will lose. That’s good for us, the management. [If] they think there is profit… they will try to relax. Now I’m telling them this is a loss [making] project…but you will have to complete [it] on time. From that point, we can give some benefit to the company.

Ian’s use of negative emotions for control is evident in employee experiences. Although Ian intended to promote employee productivity, employees feel stressed due to negative emotional experiences. The spread of negative emotions has caused employees to fear Ian instead of being productive. Some employees feel “scared” to approach Ian to speak about issues. Vivian, a female site supervisor, explains that Ian is “nothing special” and “doesn’t look after” the employees. She explains that she feels safer speaking to Charles (low Mach) than Ian (high Mach).

If we have a problem, we go to the site manager [Charles]. We can’t go above that. Honestly, we’re scared to. Because we don’t know what sort of response we’ll receive… [Ian] never sits with us and talks normally… labourers can’t speak to the top [management]. When you look at the office, there are glass rooms, and the bosses are in those rooms… [But] if he is too flexible, then we won’t learn either.

Because of their fear, some employees avoid sharing their issues with Ian. In contrast, other employees are more expressive with their emotions. Anton, a high Mach employee, explains that he “informed” his problems even if it means that it will “backfire” on him. He notes that it is not in his personality to remain calm when there is unfairness towards people. Anton’s interview was emotionally laden; he expressed his anger and dissatisfaction with Ian’s leadership.

I’ve informed my problems… I’m very surprised how [others] can bear it all and remain silent. I just can’t do that…. even if I [complain about] them…it will backfire on me…if someone stands up for unfairness, they will most likely not work next year. [Top management has] made the right environment for themselves… out in the open, they are nice and friendly, you feel happy to work, but there are things beneath the surface…

Anton explains that people in leadership positions act “friendly” to ensure the workers are “happy”. But he notes that in reality, it is a façade. The interviews suggest that high Mach employees may be more vocal and expressive about their negative emotional experiences in the workplace than low Mach employees. Other low Mach interviews noted the ability to cope with or turn a blind eye to negative emotional experiences. Chris, a low Mach employee, explains that his company does not pay him well, but the leader (Uday, high Mach) has high expectations and even shouts in meetings. Chris note feeling helpless.

Take so much work out of me; they don’t pay me well, I have so much pressure… They shout in meetings. There is nothing [we can] do…

Mike, a low Mach employee, also explains that Uday expresses many negative emotions in the workplace. Mike further notes that negative emotional experiences are “normal” in the construction industry, but he believes leaders should control their emotions.

He gets angry and cruel. It is normalised in the construction field because when he gets pressure…he sends it down to us below…A leader should control their anger because, honestly, there are so many reasons to get angry, but that should be handled.

Likewise, Claire notes, "[employees] respect leaders, and listen to them because respect is always in our nature”. She explains that Uday can get “angry”, but she “obeys” his rules because he is her leader. Interviews show that Uday’s expression of negative emotions is causing employees to feel negative emotions. During the interviews, most employees felt scared to speak openly about Uday. Most employees came across as timid and afraid to voice their genuine emotions.

#### Low Mach Leader and (High and Low) Mach Employee

This section explores the dyadic relationships between low Mach leaders and high/low Mach employees. The interviews suggest that dyadic relationships with more time to develop would experience increased positive emotions together than negative ones. Low Machs, Stuart and Bernard have over a four-year relationship and have worked in the construction industry for over ten years. Stuart explains that he trusts Bernard and gives him a promotion because he has the capabilities.

I guided [Bernard], and I gave him a full workload, and he managed to do everything… Then I gave him a promotion to project manager…Bernard has the capacity and talent; ordinary people have to spend time and gather experience; with time and age, they will get that.

Other employees did not appreciate this. Ivon (high Mach) has worked with Stuart for two years but has worked in the construction industry for over ten years. He explains feeling like a second fiddle to Bernard and despises that Stuart prioritises some employees over others. Throughout the interview, Ivon revealed various negative feelings towards Stuart. However, younger employees are keener to work with their leaders, regardless of how they are treated. Aziz explains that his low Mach leader (Thomas) guides him skilfully, and he feels safe speaking to Thomas without fear. Aziz may be optimistic about his relationship with Thomas because he is new to the construction industry (two years) and willing to learn.

They are guiding us well. If we have any issues, we can talk with them; they have given us the freedom to speak with them; without any hesitation, they allow us to speak to them. That helps us motivate ourselves… [And] correctly do the work.

However, like Ivon, high Mach employees in Thomas’s site are also more cautious about their relationships. Brian, the high Mach employee, works under the supervision of Thomas. Brian explains that although Thomas is the site leader, he relies not only on Thomas for guidance. He notes that people have good and bad, and he is selective in placing his trust in one person.

Mr Thomas is the top person… [But] I tend not to look up to one person, because every leader has positive things and negative things… I try to observe my seniors and even juniors…I try to figure out their good and bad qualities, which I should avoid and take. [I don’t rely] on one person in my life, that is my strategy.

The interview accounts suggest that low Mach leaders prioritise some employees over others, which employees do not appreciate. However, older and more experienced employees are more likely to feel negative emotions due to leaders’ prioritisations than younger and less experienced employees who are willing to acquire knowledge regardless of negative experiences. Low Mach leaders may prioritise some employees with whom they have developed a stronger relationship over time. However, high Mach employees would address their relationships with caution and a sense of distrust even if their relationship is developed.

## Implications

There are several important theoretical and practical implications arising from this study. In the following sections, we will discuss the implications for theory and practice.

### Theoretical Implications

This study highlights the complex and multi-dimensional nature of emotional processes in Machiavellian leadership. Despite, despite the empirical studies on emotions and Machiavellian leadership (Austin et al., 2014; Stradovnik & Stare, 2018;), there is little evidence on emotions across levels. This study explored emotions in Machiavellian leadership from within-person to relational levels by considering both leader and employee experiences. While research has studied the emotions of Machiavellians (Austin et al., 2007) and emotions in leadership (Silard & Dasborough, 2021; Tee, 2015), these studies have primarily drawn on quantitative methods or remained at a conceptual level (Judge et al., 2002; Puthillam et al., 2021). The current study has considered the relational nature of emotions (Humphrey et al., 2016; Tee, 2015) and leadership (Uhl-Bien & Ospina, 2012) and drawn on qualitative methods to explore the emotional processes in Machiavellian leadership. The emotional experiences of Machiavellians presented in this research have important implications for theory development.

#### Explicit Emotion Regulation and Emotion Contagion

Emotion regulation helps individuals navigate their emotional experiences, whether to “resist temptations or overcome fears” (Braunstein et al., 2017, p. 1545). There are various emotion regulation strategies. From a high-level classification, emotion regulation can be differentiated by explicit and implicit forms (Braunstein et al., 2017). Explicit emotion regulation is the conscious change in one’s emotions. Implicit emotion regulation is more automatic (Braunstein et al., 2017). The interviews with Machiavellians in this study suggest various facets of explicit emotion regulation operating at within-persons and relational levels. The interviews show that Machiavellian leaders (for example, Ian and Thomas) explicitly regulate their emotions because they believe that expressing emotions is a sign of weakness—that leaders in the construction industry should be “strong”. This explicit form of emotion regulation is at a within-person level.

Empirical studies have noted that explicit emotion regulation can also occur at relational levels (Barsade et al., 2018; Tee, 2015). In this study, the interviews with Machiavellians show two key emotional processes. First, in leader–employee interactions, Machiavellians engage in conscious emotion regulation. Second, at relational levels, Machiavellians engage in conscious emotional regulation of others, resulting in emotional contagion.

An example of explicit emotion regulation is, for instance, Chris (low Mach), noting anger towards Uday (high Mach), however, suppressing those emotions because “there is nothing [they] can do”. An example of explicit emotion regulation that results in emotional contagion (at a relational level) is, for instance, Ian inducing fear among employees to increase productivity and employees experiencing negative emotions due to Ian’s emotion regulation. Although nascent, these findings highlight the multi-dimensional relational nature of emotional processes in Machiavellian leadership, which is a novel contribution to the topic.

#### Emotional Developments of Leader–Employee Relationships

Empirical studies suggest that personality and trait elements can influence the leader’s ability to enact emotions at a relational level (Tee, 2015). Machiavellians distrust others (Christie & Geis, 1970). This study also reiterates Machiavellians’ lack of trust towards other employees. Trust is essential to the leadership process and leader–follower interactions (Agote et al., 2016). Agote et al. (2016) suggest that trust in a leader leads to positive emotional experiences, whereas distrust leads to negative emotional experiences. In this study, the leader–employee dyads illustrated that perhaps a person’s Machiavellianism influences the development of leader–follower relationships. The interviews with high Machs show distrust in others. Their distrust of others also has implications for their emotional experiences in the workplace. For instance, Anton was highly distrustful of Ian’s intentions, and because of this, Anton expressed a range of negative emotional experiences.

Low Machs also noted experiencing negative emotions, yet they were more open to learning from their experiences. For instance, Vivian explained that she was “scared” of Ian, but she believes this is an opportunity to learn the realities of different leaderships. Likewise, Chris noted feeling helpless in meetings, yet he believes that adverse experiences in the workplace can be minimised with teamwork and proper communication. Low Machs may have expressed less negative emotions to avoid conflict (Christie & Geis, 1970) and be more cooperative (McHoskey, 1999) due to their ability to be better at coping with aggression (Jones & Paulhus, 2009).

The emotional experiences in dyadic relationships also consider the aspect of ‘time’. Graen and Uhl-Bien (1995) note three stages of a relationship: stranger, acquaintance, and maturity levels based on trust, respect, and mutual obligations. Relationships evolve through these stages with time. The findings of this study note that trust in a leader may depend on the duration the dyads have had to develop their relationship. For instance, Travis and Ian have been working together for five years. Although Travis noted problems with Ian, he also mentioned that he trusted Ian to “some extent”.

In contrast, Anton and Ian have been working together for three years. Anton regards Ian with complete distrust. The duration of time these relationships have developed may influence mutual trust and emotional experiences within the relationship. A similar pattern is seen in the relationship between Ivon and Stuart (2-year relationship, less trusting and experiencing more negative emotions) compared to Bernard and Stuart (4-year relationship, more trusting and experiencing more positive emotions). Accordingly, time and Machiavellian personality may influence mutual trust in leader and employee relationships and, consequently, the evolving emotional experiences in the workplace.

#### Situational and Contextual Emotional Processes

Leadership and emotional processes are influenced by contextual elements (Humphrey et al., 2016; Tee, 2015). This study reflects the natural leadership and emotional process in the workplace. Most studies on emotions and leadership have drawn on findings from laboratory experiment settings (Barsade et al., 2018; Tee, 2015), neglecting the context and environment in which emotional processes occur. This study identifies contextual elements at organisational, industrial and national levels that may have influenced the emotional processes in Machiavellian leadership: power distance, respect culture, and industrial expectations.

First, the interviews suggest that power distance between leader and employee may influence employees’ emotional experiences. Dasborough et al. (2009) note that power distance influences the distribution of power in organisations and the extent to which followers are subjected to the leader’s emotions. Followers with fewer interactions with the leaders’ negative emotions will experience less damaging emotions (Barsade et al., 2018; Dasborough et al., 2009). However, this study suggests that followers with fewer interactions with the leader because of power distance within the workplace may experience more negative emotions. For instance, Vivian holds Ian in a negative perspective because he does not interact with her and is in a separate “glass room” that is not accessible to her. Similarly, Ivon expressed negative emotions because Stuart prioritised Bernard, causing an imbalance in power distribution and Ivon’s ability to interact with Stuart.

Second, the interviews highlight the understanding of “respect” as an essential cultural element determining how emotions are expressed in the workplace. In some contexts, followers respect leaders for their worthwhile, ‘deserving’ and outstanding qualities (Clarke, 2011). However, in Asian cultures like Sri Lanka, respect “involves acting in conformity with one’s family, social, ethical and religious norms” (Sugirtharjah, 1994, p. 1). It is a common expectation that children respect their parents; employees respect their employer; followers respect their leaders. In this study, the two female employees noted respecting their leader. Accordingly, their emotional expressions towards the leader may be controlled due to expectations of respect. It is possible that the sense of respect, in this context, is gendered and cultural.

Lastly, several Machiavellian employees highlighted that expressing negative emotions is normal in the construction industry, whereas Machiavellian leaders noted that expressing emotions is a weakness. The highly male-dominated culture may influence both perspectives within the construction industry (George & Loosemore, 2019). In male-dominated industries, individuals believe emotions can induce decision-making bias (Lerner et al., 2004). For engineers, maintaining professionalism is essential to sustain a masculine character (Liyanagamage & Fernando, 2022). As a result, they might suppress and deny emotions (Naoum et al., 2020). Leaders showing ‘empathetic’ emotions may be perceived as weak. As for employees, being in situations where the leader is angry and shouting is “normalised”, and coping with such situations would be a sign of strength.

#### Towards a Framework for Machiavellian Emotional Processes

**Fig. 1:**
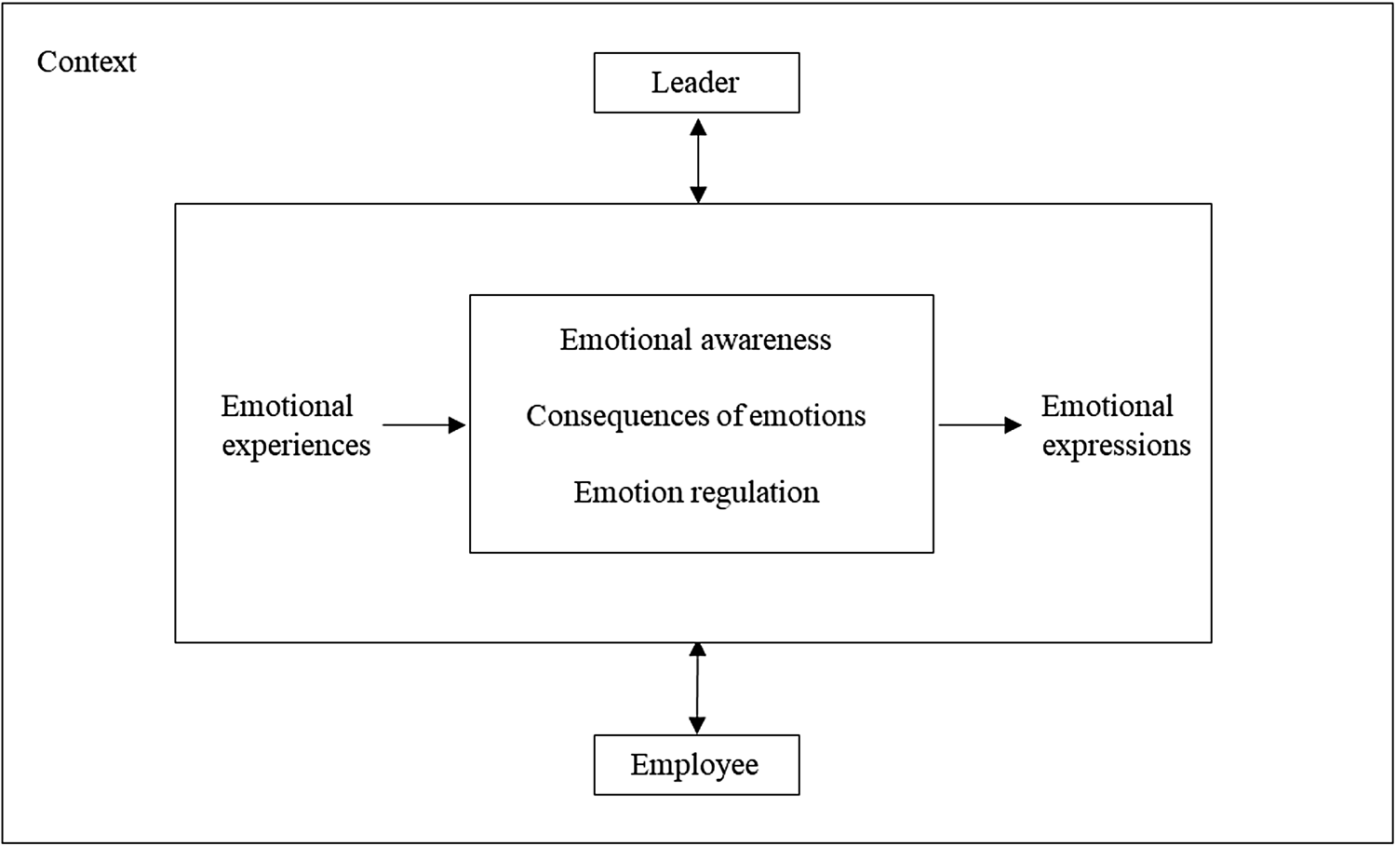
Framework for emotional processes of Machiavellians

The discussion above highlights within-persons and relational level emotional processes in Machiavellian leadership. Figure 1 presents the framework for the emotional processes of Machiavellians. We propose that initially, when an individual (high/low Mach –leader/employee) experiences emotion(s) in a situation, they would journey through a process, that is (i) emotional awareness, (ii) determine the consequences of emotions and (iii) emotion regulation before they express emotion(s).

Consider a high Mach employee (for instance, Travis) who experiences anger when their leader blames them for an action outside their responsibility. Travis may be aware of their anger and understand it is causing pain (emotional awareness). Still, displaying anger would affect their relationship with the leader (understanding the consequences of their emotions). Considering this, Travis decides to suppress their anger (emotion regulation). Their motivation to continue a positive and beneficial relationship with their leader may influence their decision to suppress negative emotions. Accordingly, various facets of Machiavellianism, such as distrust, desire for status, and amoral manipulation for goal achievement (Dahling et al., 2009), may influence how emotional processes evolve in different contextual situations and workplace relationships.

### Practical Implications

There are several practical implications of this study. First, this study suggests that time is a crucial element in developing relationships. Time has contextual relevance. Some cultures, such as Sri Lankans, respect time from a long-term orientation. These cultures consider future relationships over short-term success (Hofstede, 2011). The cultural understanding of time may frame how mutual trust in the Machiavellian leader and employee relationships develop and consequently their evolving emotional experiences. Furthermore, power distance between leaders and employees may also influence emotional experiences. These findings have cross-cultural relevance for workplaces in the Global South and for Global North hosting expatriates from the Global South.

Second, at an organisational level, explicit emotion regulation might result in emotional contagion. Machiavellian leaders may engage in fear-mongering strategies to gain control of the workplace. This has important practical implications for organisations and societies. The findings show that fear causes employees’ negative emotional experiences, potentially leading to emotional exhaustion and leaving intentions. Fear-mongering also exists at societal levels –for instance, during the COVID-19 pandemic, fear was manipulated to enforce lockdowns and control movements. Essentially, Machiavellian emotion regulation may be present at societal levels as well.

Last, the findings suggest that Machiavellians “normalise” unethical behaviours within organisations (Lyons et al., 2019). At an elementary level, this may not impact organisations. However, over time the escalation of normalising unethical behaviours may be embedded into the organisational culture. The devastating harm created by corporate scandals such as the Deepwater Horizon BP oil spill, the Enron scandal in the United States, or the misconduct in the Banking, Superannuation and Financial Services Industry in Australia bears testimony to the outcomes when unethical behaviour is normalised. This research directs organisations to be aware of who, when and why individuals normalise unethical behaviours and proactively plan to promote responsible behaviours.

## Directions for Future Research

There are several opportunities for future research to overcome the limitations of this study. First, given that this research is limited in its generalisability to other industries and cultural contexts, there is potential for this study to be replicated in different contexts. Second, this study has a disproportionate number of high and low Machiavellians. Although this may be a natural occurrence in this context, future studies could seek to balance high and low Machiavellians in their study. Third, the sample only consisted of two females. Women’s emotional experiences may be different to men’s. Future research could study the emotions of Machiavellian females or compare the emotional processes of male and female Machiavellians. Fourth, methods such as longitudinal studies may prove useful to explore the “time” dimension of evolving relationships. Finally, this study examined Machiavellian personality as a singular trait. However, it is possible that various facets of Machiavellianism, for instance, distrust or control, have different influences on within-persons and relational level emotional processes. This research is an initial endeavour to explore the emotional processes of Machiavellian leaders and employees from a relational perspective.
